# NLRP1 Functions Downstream of the MAPK/ERK Signaling via ATF4 and Contributes to Acquired Targeted Therapy Resistance in Human Metastatic Melanoma

**DOI:** 10.3390/ph14010023

**Published:** 2020-12-30

**Authors:** Zili Zhai, Prasanna K. Vaddi, Jenny Mae Samson, Tomoya Takegami, Mayumi Fujita

**Affiliations:** 1 Department of Dermatology, University of Colorado Anschutz Medical Campus, Aurora, CO 80045, USA; zili.zhai@cuanschutz.edu (Z.Z.); prasanna.vaddi@cuanschutz.edu (P.K.V.); jenny.samson@cuanschutz.edu (J.M.S.); takegami.tomoya.45z@kyoto-u.jp (T.T.); 2 Department of Veterans Affairs Medical Center, VA Eastern Colorado Health Care System, Aurora, CO 80045, USA; 3 Department of Immunology & Microbiology, University of Colorado Anschutz Medical Campus, Aurora, CO 80045, USA

**Keywords:** melanoma, NLRP1, ATF4, resistance, targeted therapy

## Abstract

The *BRAF* V600E mutation leads to constitutive activation of the mitogen-activated protein kinase (MAPK)/extracellular signal-regulated kinase (ERK) pathway and its downstream effector responses. Uncovering the hidden downstream effectors can aid in understanding melanoma biology and improve targeted therapy efficacy. The inflammasome sensor, NACHT, LRR, and PYD domains-containing protein 1 (NLRP1), is responsible for IL-1β maturation and itself is a melanoma tumor promoter. Here, we report that NLRP1 is a downstream effector of MAPK/ERK signaling through the activating transcription factor 4 (ATF4), creating regulation in metastatic melanoma cells. We confirmed that the *NLRP1* gene is a target of ATF4. Interestingly, ATF4/NLRP1 regulation by the MAPK/ERK pathway uses distinct mechanisms in melanoma cells before and after the acquired resistance to targeted therapy. In parental cells, ATF4/NLRP1 is regulated by the MAPK/ERK pathway through the ribosomal S6 kinase 2 (RSK2). However, vemurafenib (VEM) and trametinib (TRA)-resistant cells lose the signaling via RSK2 and activate the cAMP/protein kinase A (PKA) pathway to redirect ATF4/NLRP1. Therefore, NLRP1 expression and IL-1β secretion were downregulated in response to VEM and TRA in parental cells but enhanced in drug-resistant cells. Lastly, silencing *NLRP1* in drug-resistant cells reduced their cell growth and inhibited colony formation. In summary, we demonstrated that NLRP1 functions downstream of the MAPK/ERK signaling via ATF4 and is a player of targeted therapy resistance in melanoma. Targeting NLRP1 may improve the therapeutic efficacy of targeted therapy in melanoma.

## 1. Introduction

Melanoma is a highly aggressive cancer causing the majority of skin cancer deaths. Approximately 50% of melanoma patients harbor a *BRAF*, particularly V600E [1], mutation in tumors, which leads to increased expression of downstream effectors, favoring tumor survival [2]. The application of targeted therapy, including BRAF inhibitors (e.g., vemurafenib (VEM) and debrafenib) and mitogen-activated protein kinase kinase (MEK) inhibitors (e.g., trametinib (TRA) and cobimetinib), was regarded as a therapeutic breakthrough when used in patients with *BRAF*-mutated melanoma [3]. However, the downstream effectors that are suppressed by targeted therapy can be rewired during the development of adaptive or acquired resistance [2]. Therefore, uncovering the hidden downstream effectors of the mitogen-activated protein kinase (MAPK)/extracellular signal-regulated kinase (ERK) pathway can provide clues to understand melanoma biology and improve the therapeutic efficacy of targeted therapy.

It has been suggested that the inflammatory tumor microenvironment, driven by tumor-intrinsic signaling pathways, is a major checkpoint to therapeutic efficacy [4]. Tumor-promoting stromal cells, such as tumor-associated macrophages, dendritic cells, and fibroblasts, sustain tumor cell survival, immunosuppression, and drug resistance by producing diverse inflammatory mediators. We and others have demonstrated that, in addition to stromal cells, metastatic melanoma cells spontaneously release inflammatory interleukin (IL)-1β, which in turn induces stromal cells to secrete more IL-1β, thus augmenting inflammatory signaling [5,6]. As an early response to VEM, IL-1β secretion from melanoma and stromal cells is reduced [7,8]. However, following VEM treatment, IL-1β-associated signaling from inflammatory niches is enhanced and confers drug tolerance [9], suggesting that melanoma-derived IL-1β is a master regulator for drug-resistant cells.

The mechanism behind the autocrine production of IL-1β of melanoma cells is that NACHT, LRR, and PYD domains-containing protein (NLRP) inflammasomes, the cellular machinery responsible for IL-1β maturation, are constitutively activated [5]. Among a dozen NLRP proteins, NLRP1 promotes melanoma by increasing IL-1β secretion and suppresses apoptosis by inhibiting CARD-containing caspase activity [10]. *NLRP1* gain-of-function mutations have been demonstrated to contribute to constitutive inflammasome activation and IL-1β signaling in skin inflammation and cancer development [11]. Therefore, we hypothesized that NLRP1 contributes positively to the development of drug resistance in human melanoma. Recently, we demonstrated the involvement of NLRP1 in the acquired resistance of metastatic melanoma cells to the chemotherapy temozolomide (TMZ) by amplifying IL-1β signaling and activating nuclear factor κB (NF-κB) activity [12]. In the present study, we further demonstrate, for the first time, that NLRP1 functions downstream of the MAPK/ERK signaling and contributes to acquired targeted therapy resistance in melanoma. Our findings may help develop resistance mechanism-targeted inhibitors as a strategy to improve the efficacy of current therapeutics.

## 2. Results

### 2.1. The MAPK/ERK Pathway Regulates NLRP1 Expression and IL-1β Secretion through Activating Transcription Factor 4 (ATF4) in BRAF^V600E^-Mutant Human Metastatic Melanoma Cells

Investigating the early response to the MAPK/ERK pathway inhibitors can help understand and thwart drug resistance [13]. Therefore, we first determined the mechanism of NLRP1 regulation by the MAPK/ERK pathway using drug-sensitive melanoma cells.

**Figure 1:**
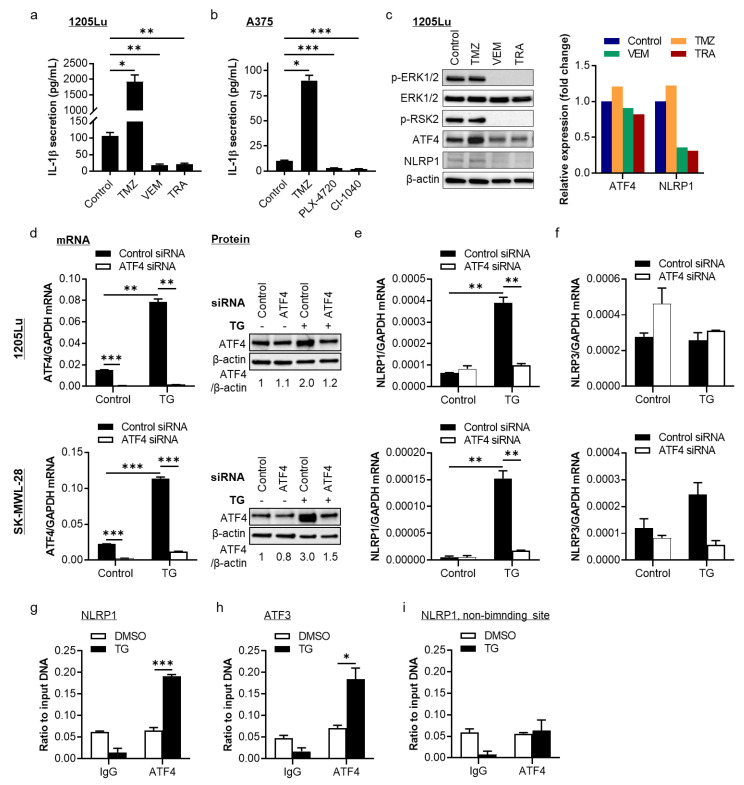
Regulation of NACHT, LRR, and PYD domains-containing protein 1 (*NLRP1*) by the mitogen-activated protein kinase (MAPK)/extracellular signal-regulated kinase (ERK) pathway through activating transcription factor 4 (ATF4). (**a**) ELISA assay of IL-1β secretion from 1205Lu cells treated with 100 µM temozolomide (TMZ), 1 µM vemurafenib (VEM), or 0.5 µM trametinib (TRA) for 24 h. (**b**) IL-1β secretion from A375 cells treated with 200 µM TMZ, 0.1 µM PLX-4720, or 0.05 µM *CI-1040* for 24 h. (**c**) Western blot analysis of ERK and RSK2 phosphorylation as well as ATF4 and NLRP1 expression in 1205Lu cells treated with dimethyl sulfoxide (DMSO, control), 100 µM TMZ, 5 µM VEM, or 0.5 µM TRA for 24 h. The band densities of ATF4 and NLRP1 were quantitated and normalized to those of the corresponding loading control β-actin (right panel). (**d**–**f**) 1205Lu (top panels) and SK-MEL-28 (bottom panels) cells were transfected with 50 nM control siRNA or *ATF4* siRNA overnight and treated with 1 µM thapsigargin (TG), an endoplasmic reticulum (ER) stress inducer, for another 18 h. (**d**) *ATF4* mRNA (left panel) and protein expression (right panel) analyzed by qRT-PCR and Western blot, respectively. The band densities of ATF4 were quantitated and normalized to those of the corresponding loading control β-actin. (**e**) *NLRP1* mRNA expression. (**f**) *NLRP3* mRNA expression. (**g**–**i**) ATF4 binds to the *NLRP1* gene promoter in metastatic melanoma SK-MEL-28 cells. SK-MEL-28 cells were treated with DMSO or 1 µM TG for 18 h, and chromatin immunoprecipitation (ChIP) assay was performed to evaluate the protein-DNA interaction, followed by qRT-PCR analysis of ATF4 occupancy at the *NLRP1* promoter (**g**). (**h**) *ATF3* was used as a positive control of ATF4 genomic targets. (**i**) The region 2 kb away from the putative binding site was used as a negative control. Representative images are shown and data expressed as the mean ± SEM, *n* = 3. * *p* < 0.05, ** *p* < 0.01, and *** *p* < 0.001.

In metastatic melanoma cells, IL-1β is released due to the constitutive activation of both NF-κB and NLRP inflammasomes [5,14,15]. While NF-κB transcriptionally regulates pro-IL-1β production, NLRP inflammasomes are involved in the maturation and secretion of this pleiotropic cytokine. To test the effects of VEM and TRA on IL-1β secretion, we used 1205Lu cells, which carry *BRAF^V600E^* mutation and have a relatively high *IL1B* mRNA expression, as well as IL-1β secretion among human metastatic melanoma cells (Figure S1) [5]. As shown in Figure 1a, in contrast to the effect of TMZ that significantly enhanced IL-1β secretion [12], VEM and TRA displayed an opposite effect and abrogated IL-1β secretion. Similar effects were observed in *BRAF^V600E^*-mutant A375 cells treated with TMZ, the BRAF inhibitor PLX-4720, and the MEK inhibitor CI-1040 (Figure 1b). Western blot data show that a single dose of VEM and TRA suppressed ERK phosphorylation and NLRP1 protein expression, whereas TMZ increased NLRP1 protein expression [12] (Figure 1c and Figure S2). These data suggest that the MAPK/ERK pathway regulates NLRP1 protein expression and IL-1β secretion.

To understand how the MAPK/ERK pathway controls the NLRP1/IL-1β, we investigated the ribosomal S6 kinase (RSK)/activating transcription factor 4 (ATF4) axis. RSK family proteins are known to regulate multiple cellular functions. RSK2, activated by ERK1/2, translocates to the nucleus and phosphorylates several transcriptional factors, including ATF4 [16]. Western blot data show that a single dose of VEM and TRA suppressed RSK2 phosphorylation and ATF4 protein expression (Figure 1c and Figure S2). In contrast, TMZ had no such effect. Interestingly, the promoter region of human *NLRP1* has a binding motif for ATF4 (Figure S3). These data indicate a role for the RSK2/ATF4 as a link between the MAPK/ERK pathway and NLRP1/IL-1β.

To understand the relationship between the MAPK/ERK pathway and NLRP1, we tested whether ATF4 works upstream of NLRP1 expression in metastatic melanoma cells. As a transcriptional factor, ATF4 regulates downstream gene expression in response to endoplasmic reticulum (ER) stress [17]. D’Osualdo et al. reported that NLRP1 is upregulated in human cervical cancer HeLa cells under ER stress conditions [18]. Therefore, we evaluated the regulation of NLRP1 by ATF4 using 1205Lu and another *BRAF^V600E^*-mutant cell line, SK-MEL-28 (Figure 1d–f). We silenced *ATF4* using siRNA in cells and treated them with an ER stress inducer, thapsigargin (TG), to induce ATF4 expression (Figure 1d and Figure S4). In the presence of TG, both *ATF4* and *NLRP1* were upregulated at mRNA levels (Figure 1d,e, respectively), suggesting that NLRP1 expression responds to ER stress activation in melanoma cells. However, *ATF4* knockdown inhibited TG-induced *NLRP1* gene transcription (Figure 1e). As a nontarget gene control, *NLRP3* gene expression was evaluated and found to be unchanged by *ATF4* knockdown (Figure 1f). These results suggest that NLRP1 lies downstream of the ER stress signaling cascade in melanoma cells, possibly through ATF4 regulation.

Next, to evaluate whether this enhanced ATF4 expression by TG has a direct regulatory effect on NLRP1, we tested the direct binding of ATF4 to the promoter region of *NLRP1* gene by protein–DNA interaction chromatin immunoprecipitation (ChIP) assay. We found increased recruitment of ATF4 to the *NLRP1* promoter upon TG treatment, while no *NLRP1* DNA enrichment was found in the negative IgG control group (Figure 1g). *ATF3*, a positive control of ATF4 target genes, was found to interact with ATF4 protein under the same conditions (Figure 1h). The region 2 kb away from the putative binding site on the *NLRP1* promoter was used as a negative control and showed no interaction with ATF4 protein (Figure 1i). These data demonstrate that ATF4 is an activator of the *NLRP1* gene in metastatic melanoma cells, especially under ER stress conditions.

### 2.2. The MAPK/ERK Pathway Shows an RSK2-Dependent Regulation of NLRP1 Gene Promoter Activity and Protein Expression in Metastatic Melanoma Cells

**Figure 2:**
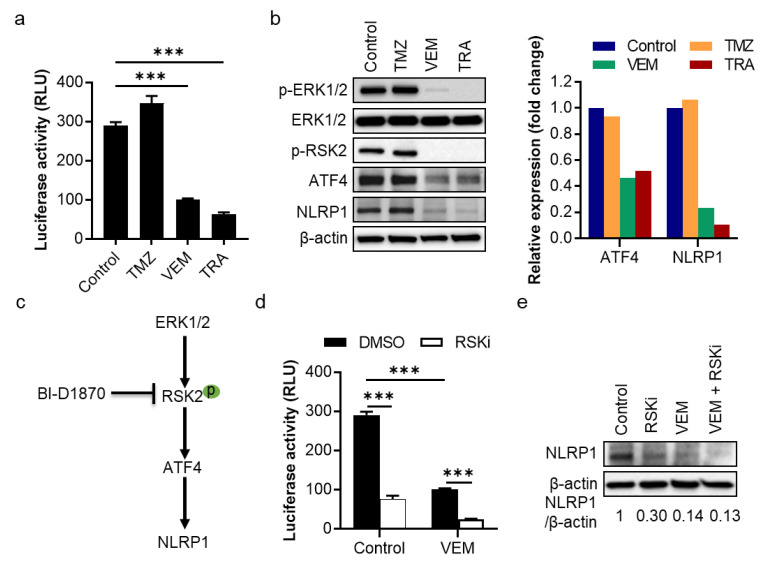
Effects of VEM, TRA, and the RSK inhibitor on *NLRP1* gene promoter activity and protein expression in SK-MEL-28 cells. (**a**) Around ~1000 bp *NLRP1* promoter region was cloned into a luciferase reporter vector and transfected into cells overnight, and cells were treated with 100 µM TMZ, 1 µM VEM, or 0.1 µM TRA for 24 h before measuring luciferase activity. (**b**) Western blot analysis of ERK activation, p-RSK2, ATF4, and NLRP1 expression in cells treated with inhibitors for 24 h. The band densities of ATF4 and NLRP1 were quantitated and normalized to those of the corresponding loading control β-actin (right panel). (**c**) Hypothesized regulation of NLRP1 by the ERK pathway through RSK2. Arrows indicate the direction of signal transduction; bar stands for inhibition; and “p” in green oval represents phosphorylation. (**d**) *NLRP1* promoter luciferase activity in cells treated with 1 µM VEM and/or 10 µM BI-D1870, an RSK inhibitor (RSKi), for 24 h. (**e**) Western blot analysis of NLRP1 expression in cells treated with 10 µM BI-D1870 and/or 1 µM VEM for 24 h. The band densities of NLRP1 were quantitated and normalized to those of the corresponding loading control β-actin. Representative images are shown and data expressed as the mean ± SEM, *n* = 4. *** *p* < 0.001.

Next, we tested whether the MAPK/ERK pathway regulates the *NLRP1* gene by measuring *NLRP1* promoter activity in SK-MEL-28 cells treated with a single dose of VEM or TRA. We found that both inhibitors significantly reduced *NLRP1* promoter activity (Figure 2a). In agreement with these findings, Western blot analysis shows that NLRP1 protein expression was also significantly inhibited by VEM and TRA (Figure 2b and Figure S2). In contrast, the drug, TMZ, had no such effects on *NLRP1* promoter activity and its protein expression.

To test whether NLRP1 is regulated by the MAPK/ERK pathway through RSK2, we used the RSK inhibitor, BI-D1870, to inhibit RSK2 activity (Figure 2c). Luciferase reporter activity shows that the RSK inhibitor significantly reduced *NLRP1* promoter activity and had a synergistic effect with VEM (Figure 2d). Western blot analysis confirmed the synergistic inhibition of NLRP1 expression by the RSK inhibitor and VEM (Figure 2e and Figure S5). This synergistic inhibitory effect on NLRP1 protein expression was also confirmed in A375 and 1205Lu cells (Figure S6). These data indicate an RSK-dependent regulation of ATF4/NLRP1 by the MAPK/ERK signaling pathway in drug-sensitive cells.

### 2.3. VEM- and TRA-Resistant Melanoma Cells Show Increased IL-1β Secretion, Upregulation of ATF4 and NLRP1 Gene Expression, and Downregulation of MITF/AXL Ratio

**Figure 3:**
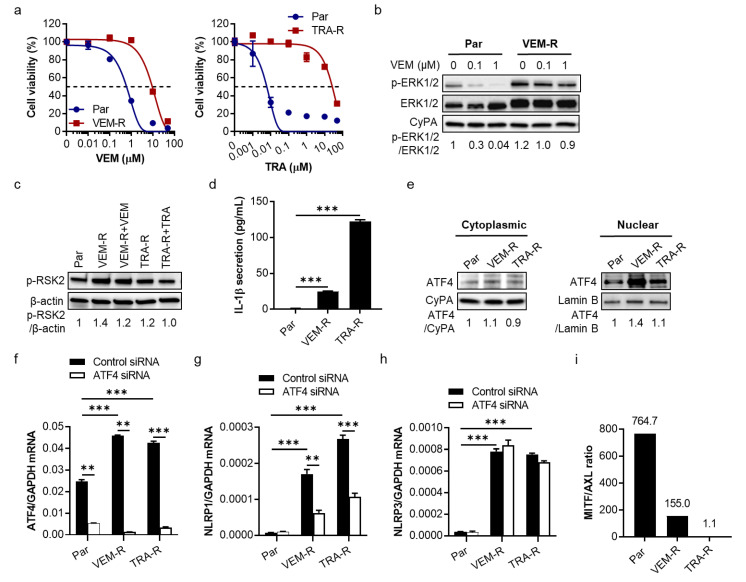
Increased IL-1β secretion, upregulated gene expression of *ATF4* and *NLRP1,* and decreased *MITF*/*AXL* ratio in VEM- and TRA-resistant SK-MEL-28 cells. (**a**) Generation of VEM-resistant (VEM-R; **left panel**) and TRA-resistant (TRA-R; **right panel**) cells by growing cells in the drug-containing medium for 2 months. An increase in 50% inhibitory concentrations (IC_50_; dashed line) indicates drug resistance. Par, parental cells. (**b**) Recovered ERK1/2 activation in VEM-R cells treated with 0.1 or 1 µM VEM for 24 h. CyPA, cyclophilin A as a loading control. (**c**) Western blot analysis of RSK2 phosphorylation in VEM-R and TRA-R cells treated with 1 µM VEM and 0.5 µM TRA, respectively, for 24 h. (**d**) ELISA assay of IL-1β secretion from parental and resistant cells. (**e**) Western blot analysis of intracellular localization of ATF4 in parental and resistant cells. Cytoplasmic and nuclear fractions of cells were isolated and assayed for ATF4 expression with CyPA and Lamin B used as markers for cytoplasmic and nuclear proteins, respectively. (**f**–**h**) qRT-PCR analysis of *ATF4*, *NLRP1*, and *NLRP3* expression, respectively, in parental and resistant cells transfected with 50 nM control siRNA or *ATF4* siRNA overnight. (**i**) The ratios of *MITF*/*AXL* gene expression in parental and MAPK inhibitor-resistant SK-MEL-28 cells. In (**b**,**c**,**e**), the band densities of p-ERK1/2, p-RSK2, and ATF4 were quantitated and normalized to those of the corresponding total ERK1/2 (**b**), loading controls β-actin (**c**), CyPA or Lamin B (**e**). Representative images are shown and data expressed as the mean ± SEM, *n* = 3. ** *p* < 0.01 and *** *p* < 0.001.

To understand the role of NLRP1 in targeted therapy resistance, we generated SK-MEL-28 cells resistant to VEM and TRA, indicated by increased IC_50_ values (Figure 3a), and verified reactivation of ERK (Figure 3b and Figure S7) and RSK2 (Figure 3c and Figure S8) in these cells. We examined how IL-1β signaling and ATF4/NLRP1 pathways were altered in these drug-resistant cells. Compared to other metastatic melanoma cells, SK-MEL-28 cells secrete extremely low to undetectable levels (<1.9 pg/mL) of IL-1β; however, both VEM- and TRA-resistant SK-MEL-28 cells displayed significantly increased IL-1β secretion (Figure 3d). The elevated levels of IL-1β secreted from these SK-MEL-28 cells indicate that NLRP inflammasomes are activated in resistant cells, leading to cellular IL-1β processing and release into the extracellular space. NLRP1 and NLRP3 are two key NLRP family members for IL-1β secretion in melanoma [12,19]. Since increased nuclear ATF4 protein (Figure 3e and Figure S9) and *ATF4* gene (Figure 3f) expression were seen in drug-resistant cells, we determined ATF4-regulated *NLRP1* gene expression. *NLRP1* expression was significantly upregulated at mRNA levels in drug-resistant cells but suppressed by knockdown of *ATF4* (Figure 3g). By comparison, *NLRP3* gene expression was upregulated in drug-resistant cells but remained unchanged with *ATF4* knockdown (Figure 3h), confirming that *NLRP1* but not *NLRP3* is regulated by ATF4, though both *NLRP1* and *NLRP3* were upregulated in drug-resistant cells. It has been reported that MAPK inhibitor-resistant SK-MEL-28 cells have a decreased expression ratio of melanocyte-inducing transcription factor (MITF) to AXL receptor tyrosine kinase, a marker of targeted therapy resistance in melanoma [20], which is possibly driven by increased ATF4 expression [21]. We confirmed the low *MITF*/*AXL* ratio by analyzing mRNA levels in drug-resistant cells (Figure 3i and Figure S10), which seems to correlate with IL-1β secretion inversely (Figure 3d). Interestingly, when we examined the relationship between *MITF*/*AXL* ratio and *IL1B* using 18 human melanoma cells without drug treatment, we found a strong inverse correlation between the *MITF*/*AXL* ratio and *IL1B* gene expression (Figure S11). Together, these data suggest that the MAPK/ERK pathway inhibitor-resistant SK-MEL-28 cells present with an increase in *ATF4*, *NLRP1*, and IL-1β secretion and a decrease in *MITF*/*AXL* ratio.

### 2.4. The cAMP/PKA Pathway Is a Regulator of ATF4/NLRP1 in Resistant Melanoma Cells

**Figure 4:**
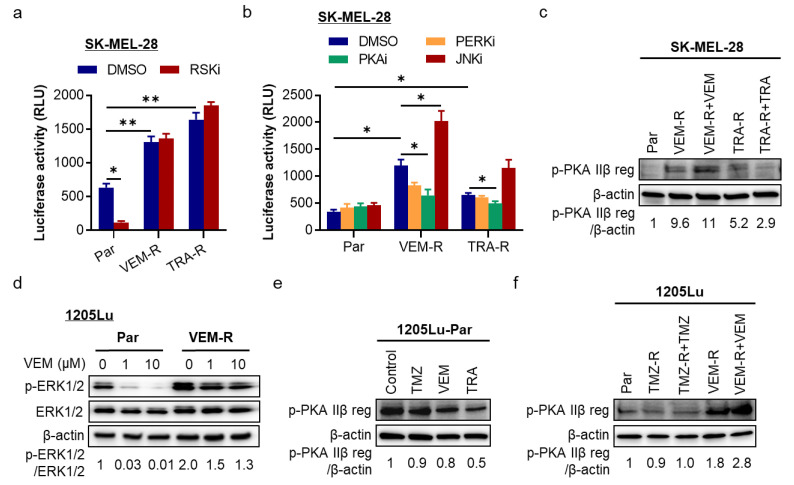
Effects of inhibiting RSK and protein kinase A (PKA) on *NLRP1* gene promoter activity in drug-resistant cells. (**a**) *NLRP1* promoter luciferase activity in parental, VEM-resistant (VEM-R), and TRA-resistant (TRA-R) SK-MEL-28 cells treated with DMSO or 10 µM BI-D1870 (RSKi) for 24 h. (**b**) *NLRP1* promoter luciferase activity in parental and resistant SK-MEL-28 cells treated with DMSO, 1 µM GSK2606414 (inhibitor of the stressor protein kinase R-like ER kinase, PERKi), 5 µM H 89 2HCl (inhibitor of PKA, PKAi), or 10 µM SP600125 (inhibitor of c-Jun N-terminal kinase, JNKi) for 24 h. (**c**) Western blot analysis of PKA IIβ regulatory subunit (PKA IIβ reg) phosphorylation in VEM-R and TRA-R SK-MEL-28 cells treated with VEM or TRA for 24 h as described in Figure 3c. (**d**) VEM-R 1205Lu cells were generated, indicated by reactivation of ERK1/2 phosphorylation. (**e**) Western blot analysis of PKA IIβ reg phosphorylation in parental 1205Lu cells treated with different drugs for 24 h as described in Figure 1c. (**f**) Western blot analysis of PKA IIβ reg phosphorylation in parental, TMZ-resistant (TMZ-R), and VEM-R 1205Lu cells treated with DMSO as control, 100 µM TMZ, or 5 µM VEM for 24 h. In (**c**–**f**), the band densities of p-PKA IIβ reg and p-ERK1/2 were quantitated and normalized to those of the corresponding loading control β-actin (**c**,**e**,**f**) or total ERK1/2 (**d**). Representative images are shown and data expressed as the mean ± SEM, *n* = 3. * *p* < 0.05 and ** *p* < 0.01.

Next, we evaluated whether the upregulated NLPR1 in resistant cells is through RSK2. Compared to parental cells, both VEM- and TRA-resistant SK-MEL-28 cells manifested significantly increased *NLRP1* promoter activity. However, unlike parental cells, increased *NLRP1* promoter activity in resistant cells remained unaffected following RSK inhibition (Figure 4a), suggesting the loss of the ATF4/NLRP1 regulation by RSK2 and a newly acquired regulation by other signaling pathways.

In fact, in addition to the ER stress and MAPK/ERK pathways, other pathways, including cAMP/protein kinase A (PKA) and MAPK/c-Jun N-terminal kinase (JNK) pathways, regulate ATF4 expression and activation [22,23]. Therefore, we investigated their effects on *NLRP1* promoter activity and found that the PKA inhibitor significantly reduced *NLRP1* promoter activity in both VEM- and TRA-resistant cells (Figure 4b). Inhibitor of the stressor protein kinase R-like ER kinase (PERK), the upstream regulator of ATF4 in ER stress, partially reduced *NLRP1* promoter activity. On the contrary, the JNK inhibitor increased *NLRP1* promoter activity in VEM-resistant cells (Figure 4b). To confirm the potential involvement of cAMP-dependent PKA in *NLRP1* gene expression, we evaluated PKA activation by determining the PKA IIβ regulatory subunit phosphorylation status in resistant SK-MEL-28 cells. Figure 4c (and Figure S8) shows an increased PKA IIβ regulatory subunit phosphorylation in both VEM- and TRA-resistant cells compared to parental cells, which was further upregulated in the presence of VEM, suggesting that the MAPK/ERK pathway cross-regulates PKA activation to affect NLRP1 expression.

We then used other VEM-resistant cells derived from 1205Lu cells (Figure 4d and Figure S12) and verified the elevated PKA IIβ regulatory phosphorylation. TMZ-resistant counterparts [12] were used as control. When parental 1205Lu cells were treated with a single dose of TMZ, VEM, or TRA, we found decreased PKA IIβ regulatory phosphorylation by signaling inhibitors, VEM and TRA, but not TMZ (Figure 4e and Figure S2), consistent with the findings in Figure 1c. However, in VEM-resistant 1205Lu cells, PKA IIβ regulatory phosphorylation was increased compared to its parental and TMZ-resistant counterparts and was further upregulated with VEM treatment (Figure 4f and Figure S8). These data support that PKA is a regulator of ATF4/NLRP1 in MAPK inhibitor-resistant melanoma cells.

### 2.5. NLRP1 Is Required for Cell Growth and Colony Formation of Targeted Therapy-Resistant Melanoma Cells

**Figure 5:**
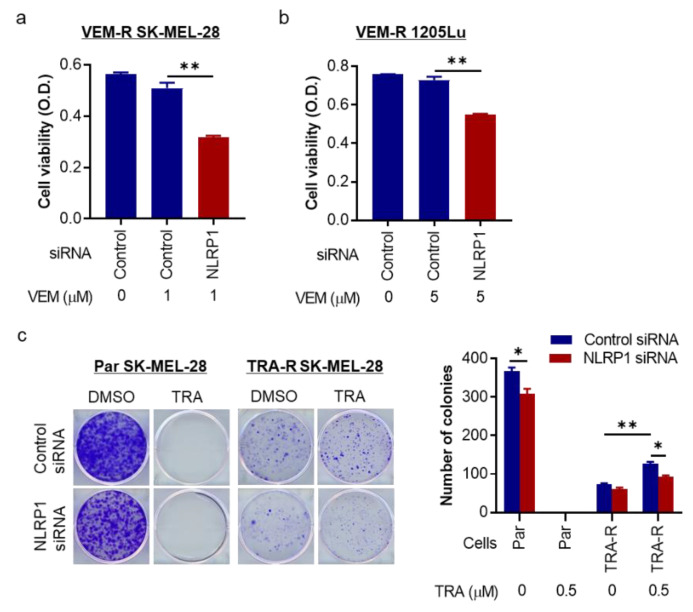
Silencing *NLRP1* slows down resistant cell growth and colony formation. (**a**) Cell viability assay of VEM-resistant (VEM-R) SK-MEL-28 cells transfected with 50 nM control or *NLRP1* siRNA overnight and treated with 1 μM VEM for 48 h. (**b**) Similarly, cell viability assay of *NLRP1* siRNA transfected VEM-R 1205Lu cells treated with 5 μM VEM for 48 h. (**c**) *NLRP1* siRNA transfected parental or TRA-resistant (TRA-R) SK-MEL-28 cells treated with DMSO or 0.5 µM TRA for 7 days, assessed for colony formation by crystal violet staining (left) and quantitated (right). Representative images are shown and data expressed as the mean ± SEM, *n* = 4. * *p* < 0.05 and ** *p* < 0.01.

Lastly, we determined the role of NLRP1 in cell growth and colony formation of MAPK inhibitor-resistant melanoma cells. Whereas VEM alone had no inhibitory effect on VEM-resistant SK-MEL-28 cell proliferation, silencing *NLRP1* decreased the proliferation of resistant cells (Figure 5a), which was confirmed using VEM-resistant 1205Lu cells (Figure 5b). The colony formation assay shows a slower growth of TRA-resistant cells compared to parental cells. However, when they were treated with TRA, the treatment left no visible colonies of parental SK-MEL-28 cells, whereas TRA-resistant cells enhanced their colony-forming ability in the presence of TRA (Figure 5c). When *NLRP1* was silenced, the knockdown inhibited the colony formation in not only parental cells without treatment, but also TRA-resistant SK-MEL-28 cells with TRA treatment (Figure 5c), indicating an essential role of NLRP1 for survival and cell growth of parental and resistant cells.

## 3. Discussion

NLRP1 participates in multiple activities in melanoma. We previously reported that NLRP1 plays a role in tumorigenesis, progression, and inflammation-associated chemotherapy resistance [10,12]. Here, we found that NLRP1-mediated IL-1β signaling is downstream of the MAPK/ERK signaling through the ATF4 regulation, further uncovering its involvement in targeted therapy resistance.

Melanoma is a type of cancer that utilizes IL-1β to shape the tumor microenvironment for its own growth. Mutational activation of *BRAF* contributes to constitutive activation of NF-κB, the key transcriptional factor that controls the production of many cytokines, including IL-1β, and is usually activated by chemotherapy and in cancer drug resistance [24,25,26]. Other pathways also regulate and augment IL-1β signaling via crosstalk with NF-κB signaling. The MAPK/ERK signaling pathway positively regulates the NF-κB pathway through multiple mechanisms, including direct phosphorylation of IκBα by RSK2 or indirect activation of IKKα/β via maternal embryonic leucine zipper kinase [27,28]. As such, it is not surprising that we observed decreased *IL1B* mRNA expression and IL-1β production in VEM and TRA-treated 1205Lu cells (Figure S13). However, inhibiting the MAPK/ERK with VEM and TRA also resulted in a decrease in IL-1β secretion (Figure 1), in concordance with previous observations [7,8]. Due to the fact that NLRP1 inflammasomes regulate not only IL-1β secretion but also NF-κB activation [10] and IL-1β production [5,10], our data suggest that the MAPK/ERK signaling modulates IL-1β signaling via the NF-κB pathway and NLPR1 inflammasomes.

ER stress is an adaptive mechanism by which cells can survive in an unfavorable environment, though it often triggers cell death upon excessive or persistent disturbance [29]. Unfortunately, this ER defense strategy can be fully used by tumor cells to survive under hostile microenvironments, such as nutrient shortage, oxidative stress, and drug pressure [30]. Sustained activation of ER stress enables tumor cells with greater tumorigenic, metastatic, and drug-resistant capacity [30,31]. We have assessed the ER stress dependence of melanoma cell growth in vitro and found that pretreatment of 1205Lu and SK-MEL-28 cells with 4-phenylbutyric acid, an ER stress inhibitor [32], sensitizes cells to TMZ and VEM treatment, respectively (Figure S14). Therefore, manipulating ER stress, including suppressing ATF4, has been regarded as an important strategy for therapeutic intervention in cancer [13,22,33,34]. Indeed, the regulation of ATF4 expression by the MAPK/ERK pathway has been recently reported [13,35]. ATF4 is a master transcriptional factor of the ER stress response, and its expression is typically upregulated in solid tumors compared to normal tissues [33]. We examined *ATF4* mRNA expression in human melanoma tissues using two publicly available microarray datasets from independent gene profiling studies [36,37] and found that metastatic melanoma tissues display higher *ATF4* mRNA level than primary melanoma (Figure S15). In agreement with this, we observed elevated levels of *ATF4* mRNA and nuclear ATF4 protein in MAPK inhibitor-resistant SK-MEL-28 cells compared to parental cells (Figure 3), suggesting that ATF4 is a critical contributor in MAPK inhibitor resistance. Recently, PERK and/or IRE1α branches of ER stress were found to enhance *NLRP1* gene expression through the cAMP response element binding protein (CREB) in HeLa and chronic myelogenous leukemia cells [18,38]. We provide further evidence that NLRP1 lies downstream of the ER stress signaling cascade, possibly through the PERK/ATF4 in melanoma cells. ATF4 transcriptionally regulates many pro-survival genes, including *HMOX1*, *BCL2*, and *MCL1*, and key autophagy genes such as *ATG5* and *ATG7* [33,35,39]. We added *NLRP1* to this pro-survival ATF4 target gene list.

**Figure 6:**
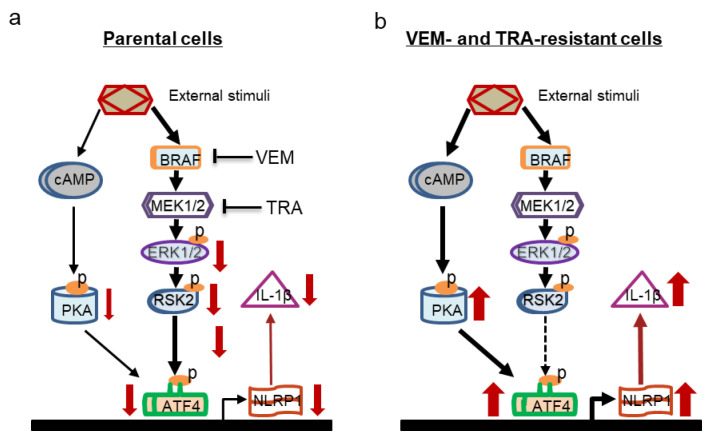
Hypothetical roles of ATF4/NLRP1 in acquired targeted therapy resistance in melanoma. (**a**) ATF4/NLRP1 in parental melanoma cells. Red arrows indicate the inhibitory effects of VEM and TRA. Bars indicate inhibition. (**b**) ATF4/NLRP1 in resistant melanoma cells. Red arrows indicate the upregulation of expression levels. A dashed line indicates a signal loss. In both a and b, the thickness of red arrows indicates relative expression levels; the thickness of black arrows indicates the relative strength of signaling pathways; and “p” in orange oval indicates phosphorylation.

NLRP1 is considered to be a tumor suppressor, but aberrant NLRP1 inflammasome activation due to gain-of-function mutations has been associated with cancers and autoimmune diseases [11,40,41], which could reasonably explain why some melanoma cell lines, if not all, are capable of secreting IL-1β. Our current work has linked NLRP1 inflammasome activation to the MAPK/ERK signaling pathway and indicated that NLRP1 inflammasome activation is implicated in the downstream signaling cascades of the activating *BRAF* mutation. In parental melanoma cells, the RSK2/ATF4 axis acts as the hub between the MAPK/ERK signaling and NLRP1; however, MAPK inhibitor-resistant cells seem to lose this signaling hub and switch to the cAMP/PKA pathway to redirect NLRP1 expression (Figure 6). In fact, there is a crosstalk between the MAPK/ERK signaling and the cAMP/PKA pathway, which are mutually exclusive [42]. In *RAS*-mutated melanoma, PKA regulates the MAPK/ERK signaling by switching BRAF to RAF-1 signaling [43]. On the other hand, active RSK inhibits PKA activity while inactive RSK interacts with PKA and sensitizes it to cAMP stimulation [39]. It has been reported that dibutyryl-cAMP, a cAMP analogue, can induce NLRP1 expression in human myeloid leukemia cells, possibly through PKA-mediated CREB activation [44]. In the current work, we verified that the regulation of the *NLRP1* gene promoter activity in MAPK inhibitor-resistant melanoma cells is via PKA. It is possible that in the reactivated, *BRAF*-mutated melanoma cells, there exists a signaling network including the ER stress, MAPK/ERK, and cAMP/PKA signaling pathways, which interact with each other and play an integral role in the self-survival of tumor cells. In this network, NLRP1 functions downstream through a shared transcriptional regulation by ATF4. Given the role of these signaling pathways in melanoma tumorigenesis and progression, downstream NLRP1 may be an important executor. This scenario could explain the tumor-promoting role of NLRP1, not only in the targeted therapy resistance observed in this study, but also in the chemotherapy resistance we observed previously [12]. These two different drug treatments activate distinct signaling pathways that converge on the ATF4/NLRP1 axis to regulate melanoma growth. This ATF4/NLRP1 axis may also be a common molecular mechanism behind cancer resistance to other therapeutic approaches.

NLRP1 is expressed at low levels in melanoma and nonmelanoma skin cancers [10,45], possibly due to its DNA hypermethylation. However, increased NLRP1 expression and activated NLRP1 inflammasomes favor drug resistance. Since the inflammatory microenvironment provides melanoma tolerance to targeted therapy [9], targeting NLRP1/IL-1β may be an important strategy to improve MAPK inhibitor efficacy. Whereas many inflammasome inhibitors are available currently, most of them target NLRP3, IL-1β, IL-1 receptor, inflammasome adaptor ASC, and inflammasome effector caspase-1 [46,47]. It has been reported that aspirin and green tea component epigallocatechin-3-gallate suppress NLRP1 expression in humans [48,49]; however, NLRP1 inhibitors so far have not been at the forefront of development. It is critical to developing such unique drugs for inflammation, cancer, and other diseases.

It is worth noting that the majority of our data were generated using SK-MEL-28 and 1205Lu cells. Due to the intertumoral and intercellular heterogeneity in human melanoma, our findings should be verified in more melanoma cell lines. Moreover, though we observed increased PKA activation in BRAF/MEK inhibitor-resistant melanoma cells, the mechanism behind PKA activation and its impact on the resistance acquisition need to be further investigated. In addition to ATF4, NLRP1 may be subject to regulation by other transcription factors. For example, ATF2 and ATF4 share the same binding cAMP response element within the *NLRP1* promoter. Our preliminary study shows that knocking down *ATF2* also reduces *NLRP1* gene expression in VEM- and TRA-resistant SK-MEL-28 cells (Figure S16). It has been reported that the PKCε-ATF2 axis is involved in melanomagenesis and BRAF inhibitor resistance [50]. Furthermore, ATF2 can be phosphorylated and activated by the MAPK/ERK pathway in collaboration with another MAPK member, p38 [51]. Therefore, it will be interesting to evaluate the regulatory role of ATF2 in NLRP1-mediated targeted therapy resistance in melanoma.

## 4. Materials and Methods

### 4.1. Chemicals

VEM, TRA, PLX-4720, CI-1040, RSK1/2/3/4 inhibitor BI-D1870, PKA inhibitor H 89 2HCl, JNK1/2/3 inhibitor SP600125, and PERK inhibitor GSK2606414 were from Selleck Chem (Houston, TX, USA). TMZ and TG, ER stress inducers, were from Sigma (St. Louis, MO, USA). These reagents were reconstituted in 100% dimethyl sulfoxide (DMSO) and stored at −20 °C in aliquots. Other chemicals and reagents were indicated elsewhere.

### 4.2. Cell Culture

Human metastatic melanoma cell lines A375 (CRL-1619) and SK-MEL-28 (HTB-72) were obtained from the American Type Culture Collection (Manassas, VA, USA) and 1205Lu cell line (1205Lu-01-0001) was purchased from Rockland (Limerick, PA, USA). Cells were routinely grown in RPMI medium 1640 (Gibco, Grand Island, NY, USA) supplemented with 10% fetal bovine serum (Gemini Bio-Products, West Sacramento, CA, USA). These cell lines have been authenticated using short tandem repeat (STR) fingerprinting by the Barbara Davis Center BioResource Core at the University of Colorado Anschutz Medical Campus and were regularly tested for mycoplasma contamination.

### 4.3. Cell Growth Inhibition

To evaluate cell’s sensitivity to VEM and TRA, melanoma cells were seeded into a 96-well plate at a density of 1–2 × 10^3^ cells per well and allowed to adhere for ~2 h before being exposed to increasing concentrations of drugs in triplicate for 72 h. Cell growth inhibition was determined using the CellTiter 96 Aqueous One Solution Cell Proliferation Assay Kit (Promega, Madison, WI, USA).

### 4.4. Generation of Acquired Resistance

Cells were plated into a 100 mm dish at a density of 1–2 × 10^5^ cells per dish and treated with increasing doses of VEM or TRA per passage for two months [52]. The starting doses of VEM and TRA were 0.05 and 0.005 µM, respectively. Acquired resistance was defined as at least a 10-fold increase of IC_50_ over that of parental cells. Resistant cells were regularly maintained in a medium with 1 (SK-MEL-28) or 3 (1205Lu) µM VEM or 0.2 µM TRA (SK-MEL-28). TMZ-resistant 1205Lu cells have been described previously [12].

### 4.5. Enzyme-Linked Immunosorbent Assay (ELISA)

A human IL-1β ELISA kit was obtained from R&D Systems (Minneapolis, MN, USA). Culture supernatants were collected for quantitating IL-1β secretion, with cell lysates for assaying intracellular pro-IL-1β levels [12].

### 4.6. siRNA Transfection

Cells seeded in appropriate culture plates at 60% confluency were transfected with 50 nM of NLRP1 siRNA (a mixture of two preselected siRNAs; Qiagen, Valencia, CA, USA) or ATF4 siRNA (a pool of three target-specific 19–25 nt siRNA; Santa Cruz Biotechnology, Santa Cruz, CA, USA), as well as their respective negative control using Lipofectamine 2000 (Invitrogen, Carlsbad, CA, USA) in OPTI-MEM1 reduced serum medium (Gibco) for 4–6 h. An equal volume of culture medium with 20% fetal bovine serum was then added.

### 4.7. Quantitative RT-PCR

RNA isolation and quantitative PCR have previously been described [12]. The primers for *IL1B*, *ATF4*, *NLRP1*, *NLRP3*, *MITF*, *AXL,* and *GAPDH* are listed in Supplemental Table S1.

### 4.8. Western Blot

As described previously [12], Western blot analysis was carried out. The primary antibodies included mouse anti-NLRP1 (Enzo Life Sciences, Farmingdale, NY, USA), mouse anti-ATF4 (Santa Cruz Biotechnology), rabbit anti-phospho-p44/42 MAPK (p-ERK1/2), rabbit anti-p44/42 MAPK (ERK1/2), rabbit anti-CyPA (Cell Signaling Technology, Danvers, MA, USA), mouse anti-phospho-RSK2, mouse anti-phospho-PKA IIβ reg, goat anti-Lamin B, and rabbit anti-β-actin (Santa Cruz Biotechnology).

### 4.9. Chromatin Immunoprecipitation (ChIP) Assay

ChIP assay of ATF4 binding to *NLRP1* gene promoter was performed using the Pierce Magnetic ChIP kit (Pierce Biotechnology, Rockford, IL, USA). Briefly, cells at 60% confluency were treated with DMSO or 1 µM TG for 18 h and cross-linked with 1% formaldehyde for 6 min, followed by quenching with 1× glycine for 5 min at room temperature. After washing, cells were lysed and nuclei digested with the MNase at 37 °C for 15 min, followed by sonication for 10 cycles at 4 °C with a 20 s pulse at 40% amplitude with a 20 s pause between each cycle. Digested chromatin was obtained by centrifugation and then split into two aliquots incubated with mouse anti-ATF4 or control IgG antibodies for 2 h. Protein A/G magnetic beads were then added and incubated at 4 °C for another 2 h. Finally, protein and RNA were removed by digestion of the eluted protein–chromatin complex with proteinase K, and DNA was purified following the manufacturer’s protocol. ChIP DNA was detected by qPCR using the Power SYBR Green PCR Master Mix with specific primers for NLRP1 and ATF3 genomic promoter regions (Supplemental Table S1).

### 4.10. Luciferase Reporter Assay

LightSwitch Promoter Reporter vector containing a 905-bp human *NLRP1* promoter region cloned upstream of the RenSP luciferase gene was from Active Motif (Carlsbad, CA, USA). Cells were transfected with the construct and an empty promoter vector using FuGENE HD transfection reagent (Active Motif) for 24 h according to the manufacturer’s instructions. Luciferase reporter signals were monitored using the Lightswitch Luciferase assay reagent (Active Motif) as per manufacturer’s protocol.

### 4.11. Clonogenic Assay

Single-cell suspensions were prepared and 1000 cells seeded into 6-well plates per well. Following treatment with TRA for 7 days, the colonies were fixed in 4% paraformaldehyde for 10 min and stained with 0.2% (*w*/*v*) crystal violet for 30 min. Digital images of the colonies were obtained using a scanning device and the colonies were counted using Image J software [53].

### 4.12. Statistical Analysis

GraphPad Prism 7 was used for testing the differences between the groups by one-way ANOVA with Bonferroni’s or Dunnett’s post-tests. A value *p* < 0.05 was considered significant.

## 5. Conclusions

Overall, this work added a new understanding of NLRP1 in melanoma biology. This is the first report to link the MAPK/ERK signaling to NLRP1 in melanoma through the ATF4 regulation. ATF4-mediated NLRP1 expression and IL-1β efflux may be the mechanistic basis for melanoma tumorigenesis and drug resistance via shaping the inflammatory tumor microenvironment. Our findings support that NLRP1 may be a promising molecular target for melanoma treatment.

## Data Availability

The data presented in this study are available in this article or associated supplementary material.

## Supplementary Materials

The following are available online at https://www.mdpi.com/1424-8247/14/1/23/s1, Figure S1: The mRNA expression levels of *IL1B* in human melanoma cell lines. Figure S2: Original blots of Figure 1c, Figure 2b and Figure 4e. Figure S3: Putative ATF4 binding site within the *NLRP1* promoter. Figure S4: Original blots of Figure 1d. Figure S5: Original blots of Figure 2e. Figure S6: Synergistic inhibition of NLRP1 protein expression by RSKi and VEM in A375 and 1205Lu cells. Figure S7: Original blots of Figure 3b. Figure S8: Original blots of Figure 3c and Figure 4c,f. Figure S9: Original blots of Figure 3e. Figure S10: *MITF* and *AXL* gene expression in MAPK inhibitor-resistant SK-MEL-28 cells. Figure S11. *IL1B* gene expression is inversely related to the expression ratio of *MITF*/*AXL* in melanoma cells as shown in Figure S1. Figure S12: Original blots of Figure 4d. Figure S13: Decreased *IL1B* mRNA expression and IL-1β production in 1205Lu cells treated with 1 µM vemurafenib (VEM) or 0.5 µM trametinib (TRA). Figure S14: Pretreatment of 1205Lu and SK-MEL-28 cells with 4-phenylbutyric acid (PBA), an ER stress inhibitor, which sensitizes cells to temozolomide (TMZ) and vemurafenib (VEM), respectively. Figure S15: Metastatic melanoma tissues display higher *ATF4* mRNA levels than primary melanoma by examining *ATF4* mRNA expression in human melanoma tissues using two publicly available microarray datasets. Figure S16: qRT-PCR analysis of *NLRP1* expression in parental and resistant SK-MEL-28 cells transfected with 50 nM control siRNA or *ATF2* siRNA overnight. Table S1: Primers used for quantitative qRT-PCR and ChIP.
