# Supplementary material for: NLRP1 Functions Downstream of the MAPK/ERK Signaling via ATF4 and Contributes to Acquired Targeted Therapy Resistance in Human Metastatic Melanoma
Source: Pharmaceuticals (Basel). 2020 Dec 30;14(1):23. doi: 10.3390/ph14010023 (PMC7823742; doi:10.3390/ph14010023)
Supplement: Supplementary file 1 [file pharmaceuticals-14-00023-s001.pdf]

Supplemental data

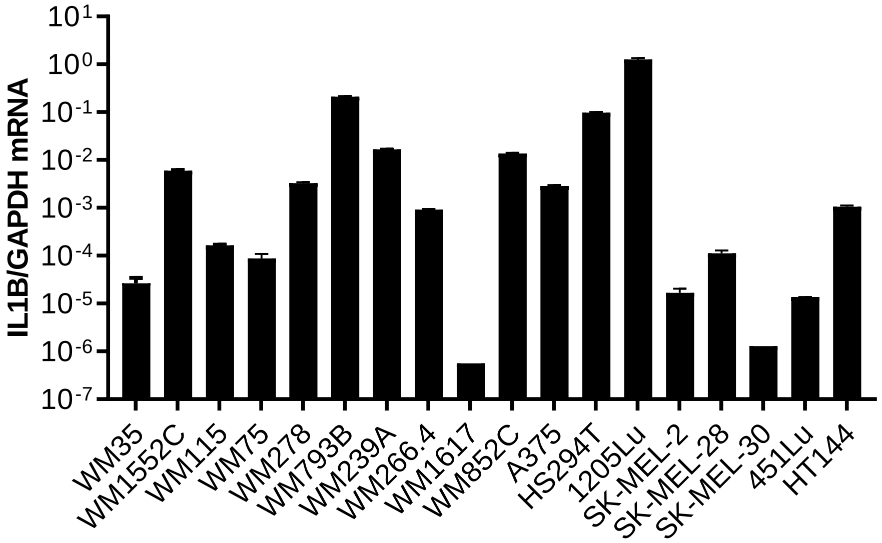

Figure S1. The mRNA expression levels of *IL1B* in human melanoma cell lines.

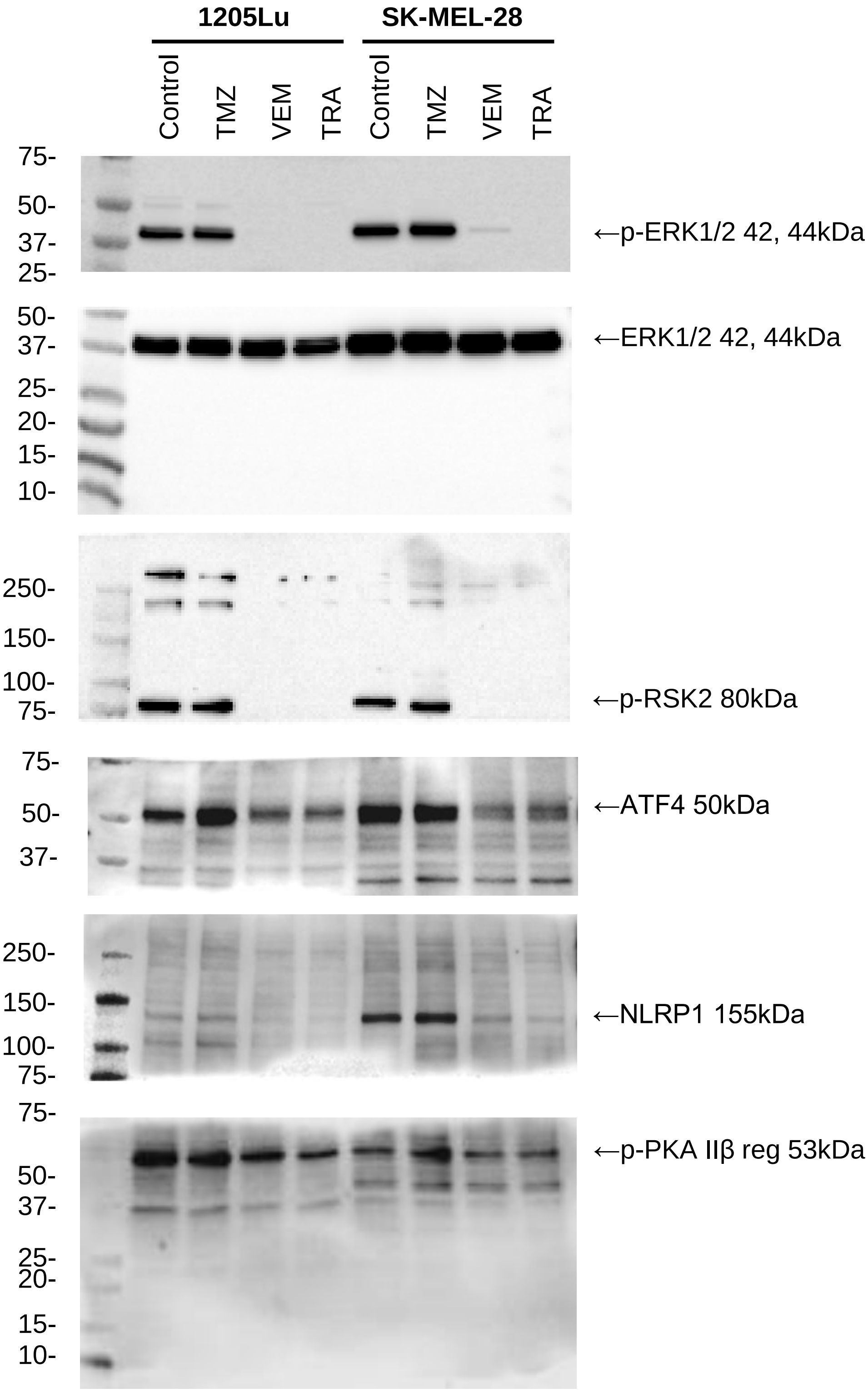

Figure S2. Original blots of Figure 1c, 2b, and 4e.

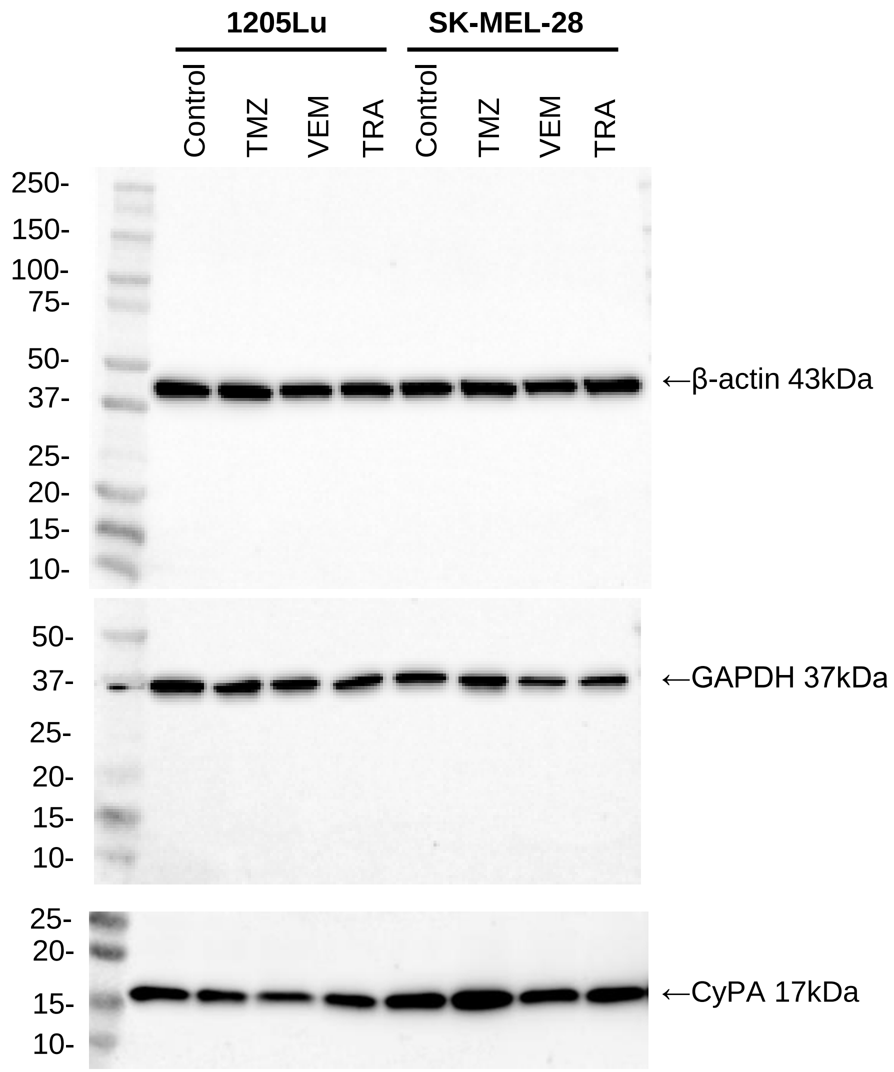

β-actin, GAPDH and CyPA were used as loading control.

Figure S2. Original blots of Figure 1c, 2b, and 4e (cont'd).

Putative ATF4 binding site
within the NLRP1 promoter

5’-G T T A C A T C A G-3’
3’-C A A T G T A G T C-5’

Figure S3. Putative ATF4 binding site within the *NLRP1* promoter.

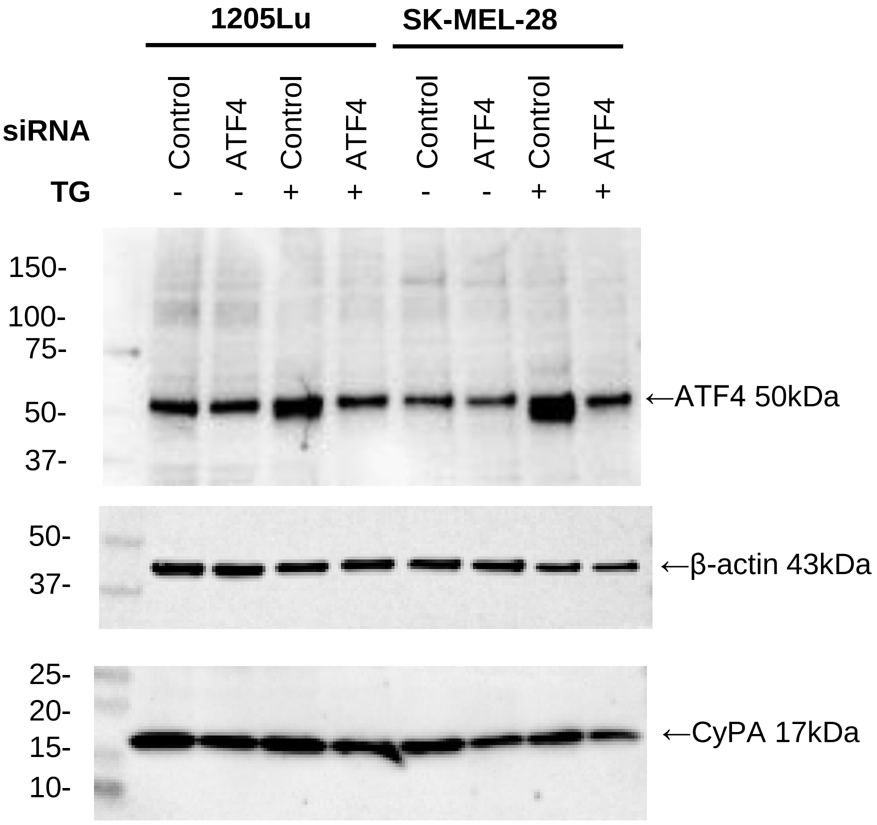

Both β-actin and CyPA were used as loading control.

Figure S4. Original blots of Figure 1d.

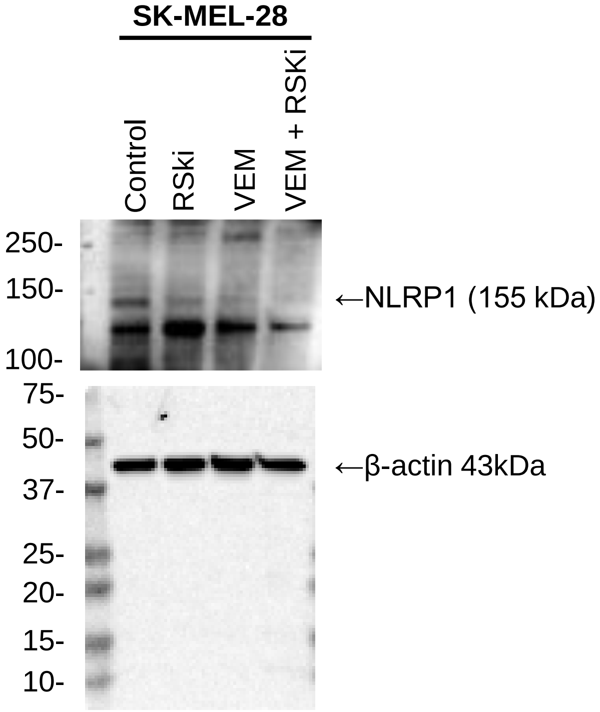

Figure S5. Original blots of Figure 2e.

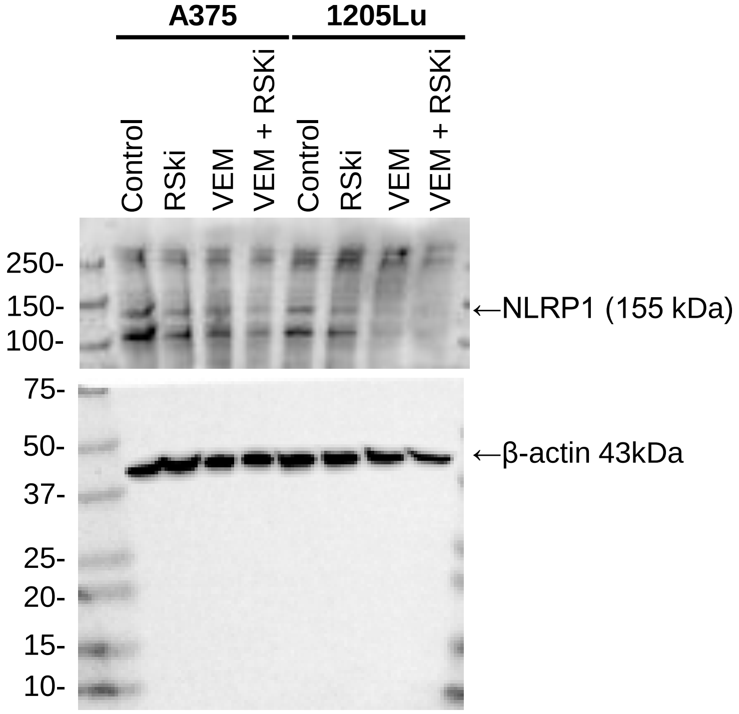

Figure S6. Synergistic inhibition of NLRP1 protein expression by RSKi and VEM in A375 and 1205Lu cells.

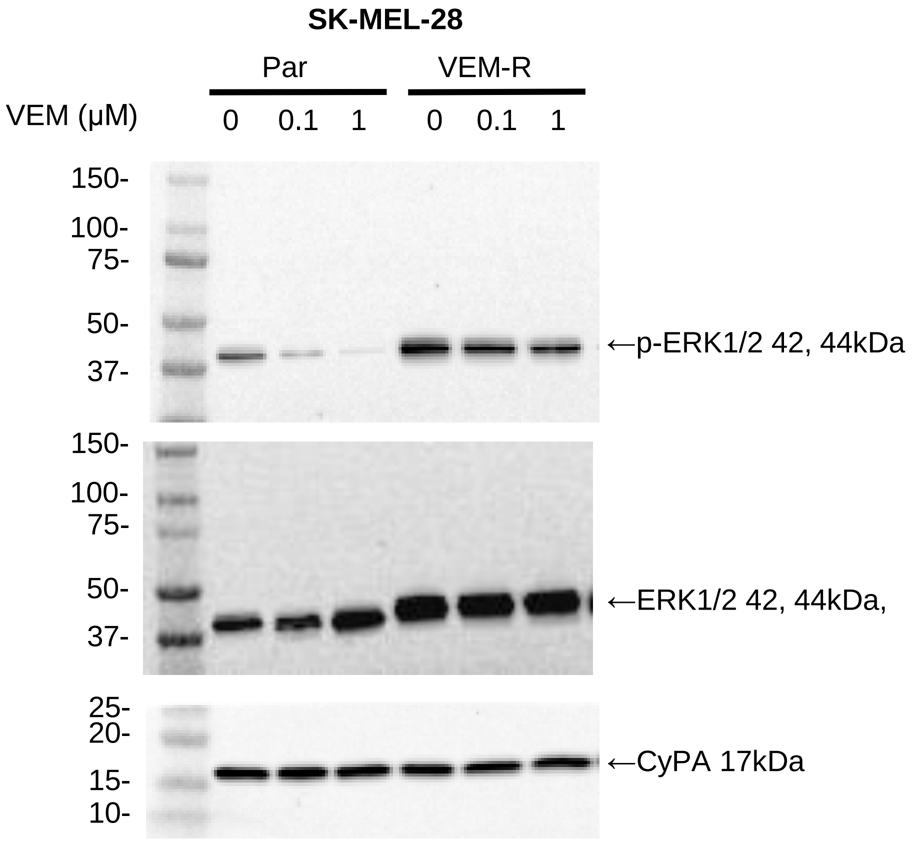

Figure S7. Original blots of Figure 3b.

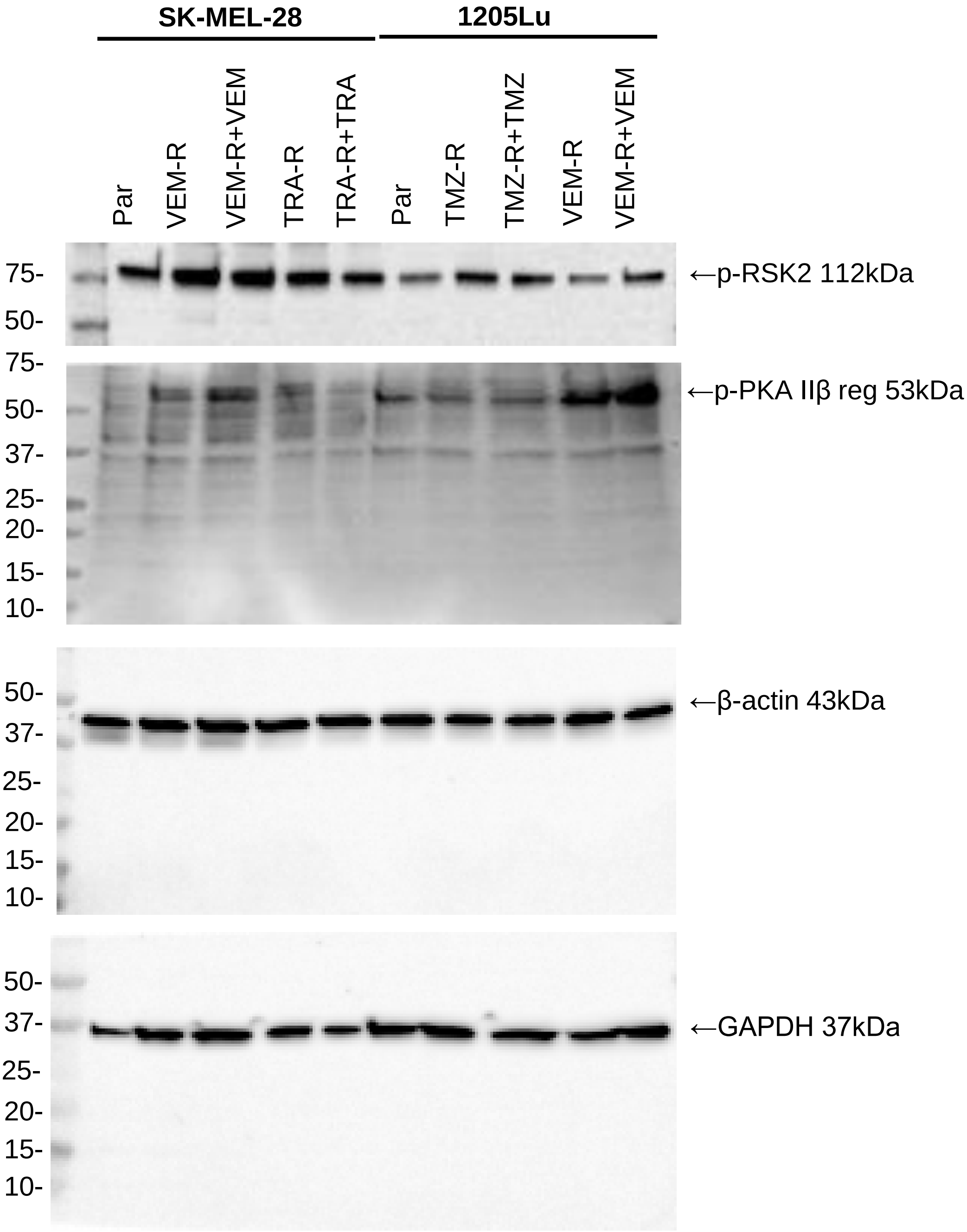

Figure S8. Original blots of Figure 3c, 4c and 4f.

Cytoplasma

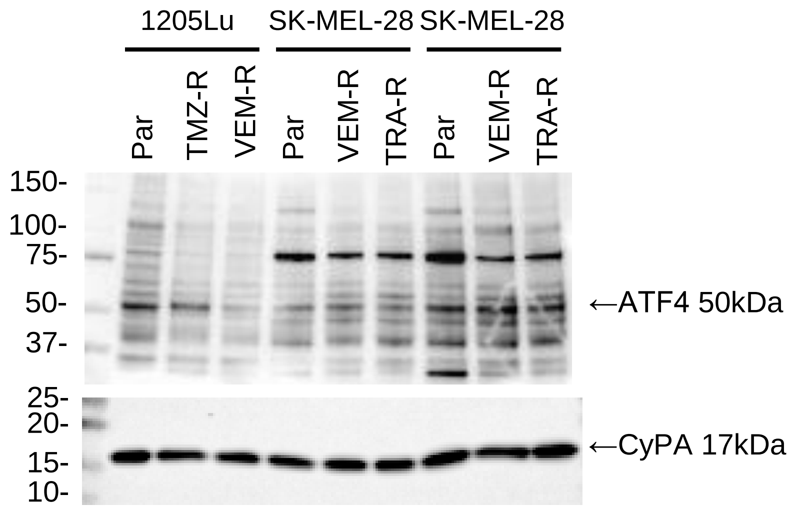

Nuclear

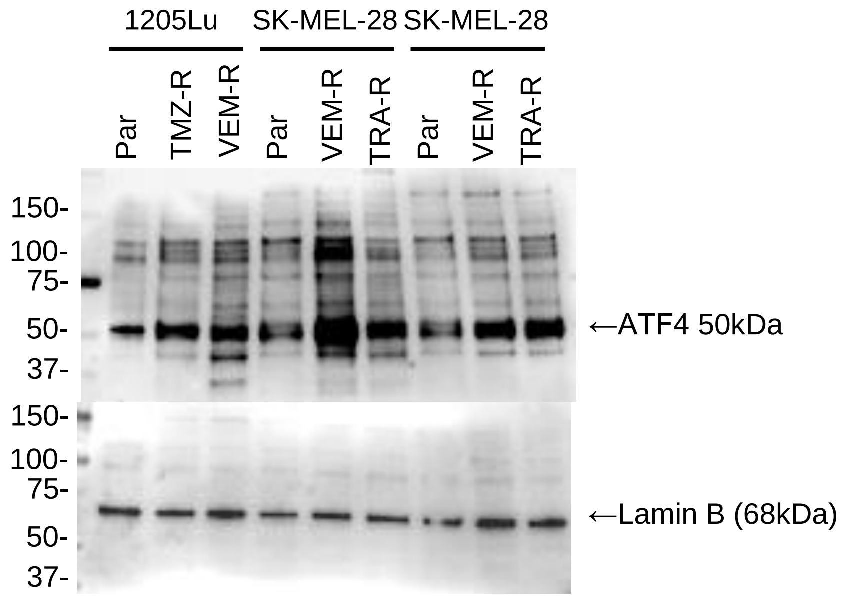

Note: Different passages of VEM-R and TRA-R SK-MEL-28 cells were tested. Left data of SK-MEL-28 cells were shown in the main text. 1205Lu data were not shown in the text.

Figure S9. Original blots of Figure 3e.

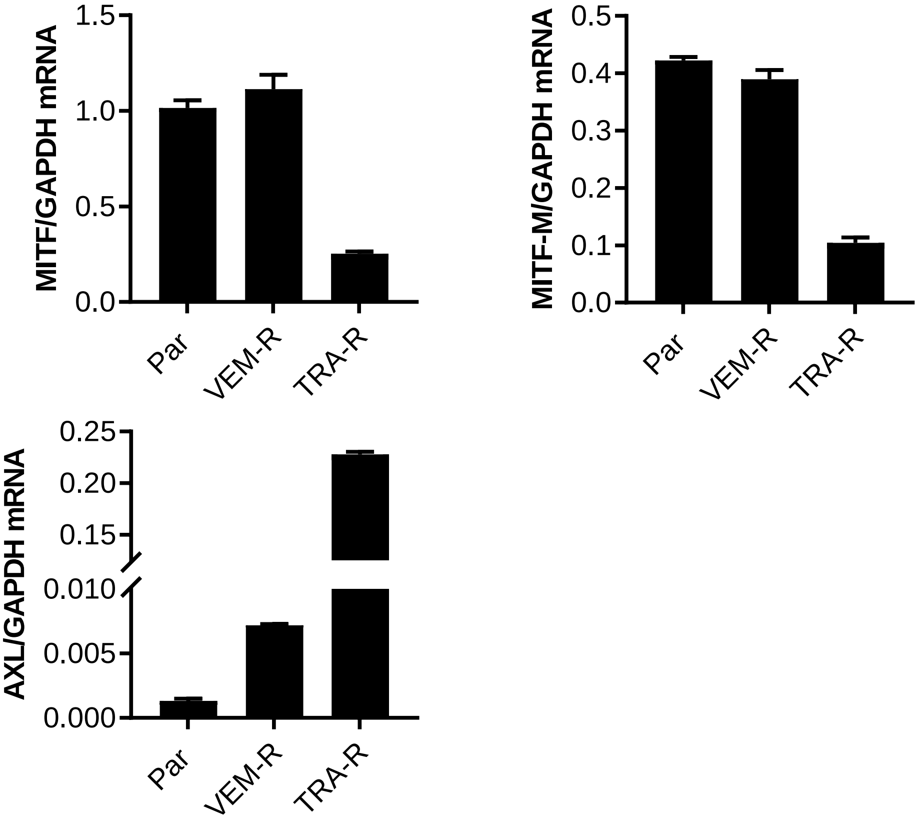

Figure S10. *MITF* and *AXL* gene expression in MAPK inhibitor-resistant SK-MEL-28 cells. *MITF-M* (MITF type M, exclusively expressed in melanocytes and melanoma cells) was also determined.

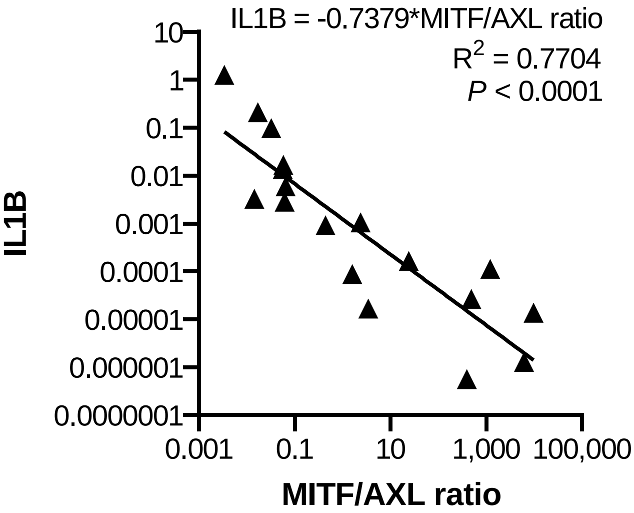

Figure S11. *IL1B* gene expression is inversely related to the expression ratio of *MITF*/*AXL* in melanoma cells as shows in Figure S1.

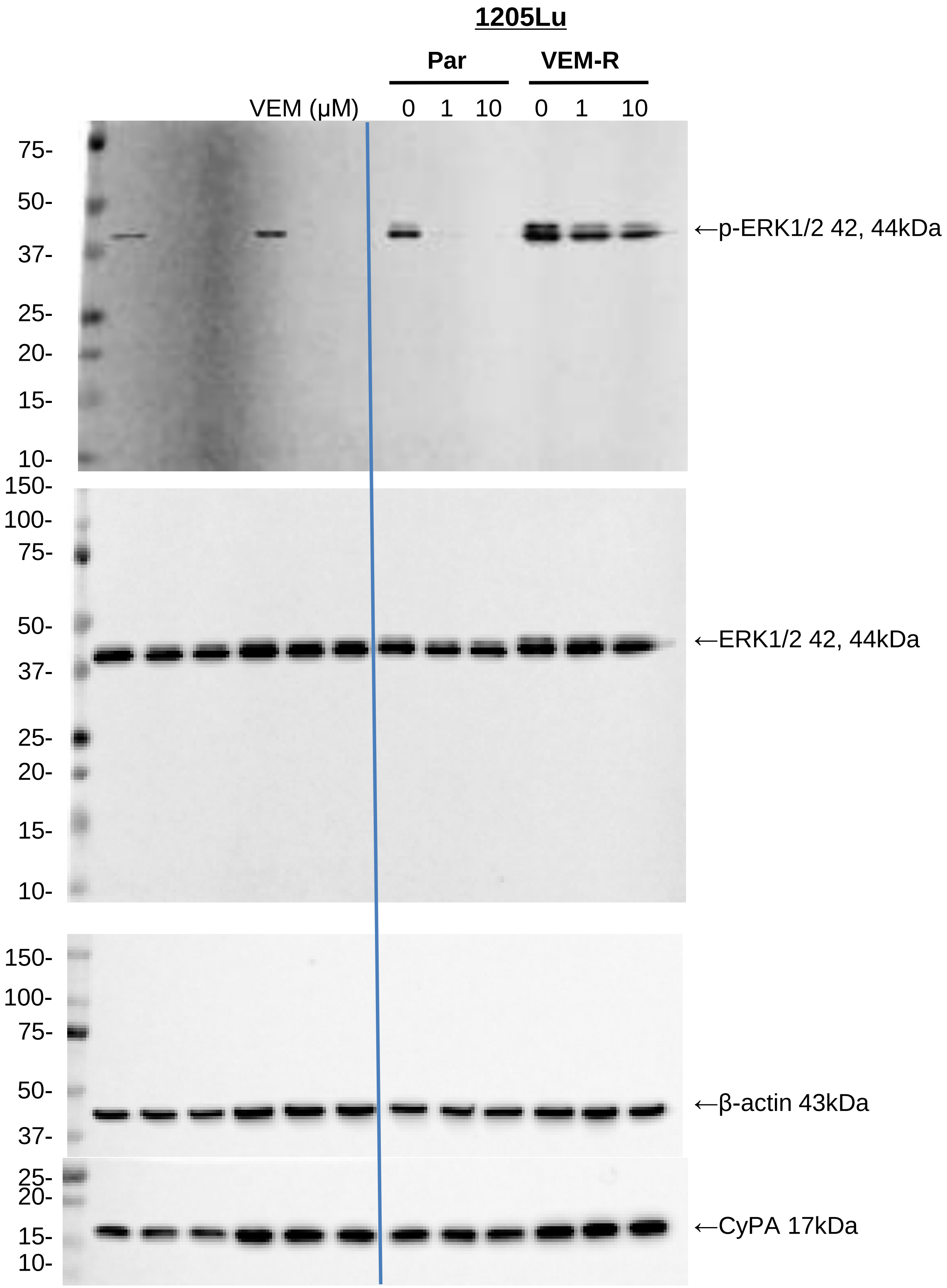

Figure S12. Original blots of Figure 4d.

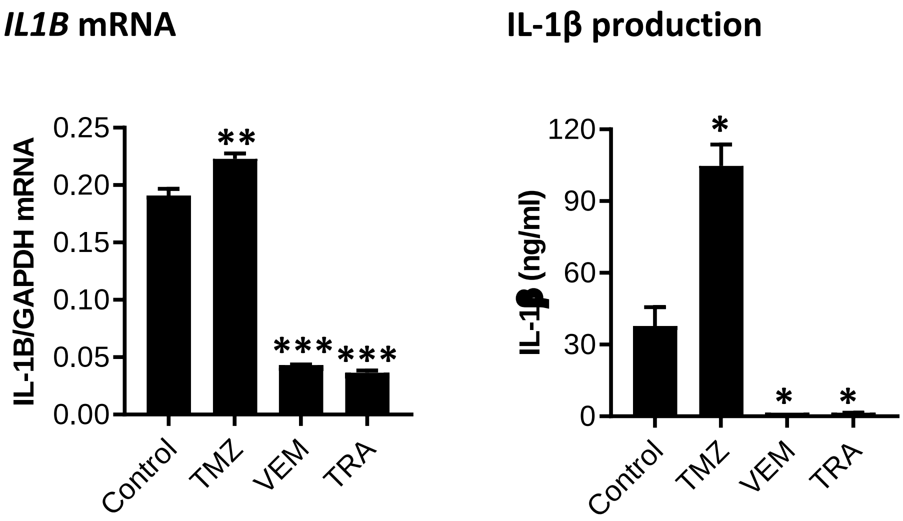

Figure S13. Decreased *IL1B* mRNA expression and IL-1β production in 1205Lu cells treated with 1 µM vemurafenib (VEM) or 0.5 µM trametinib (TRA).

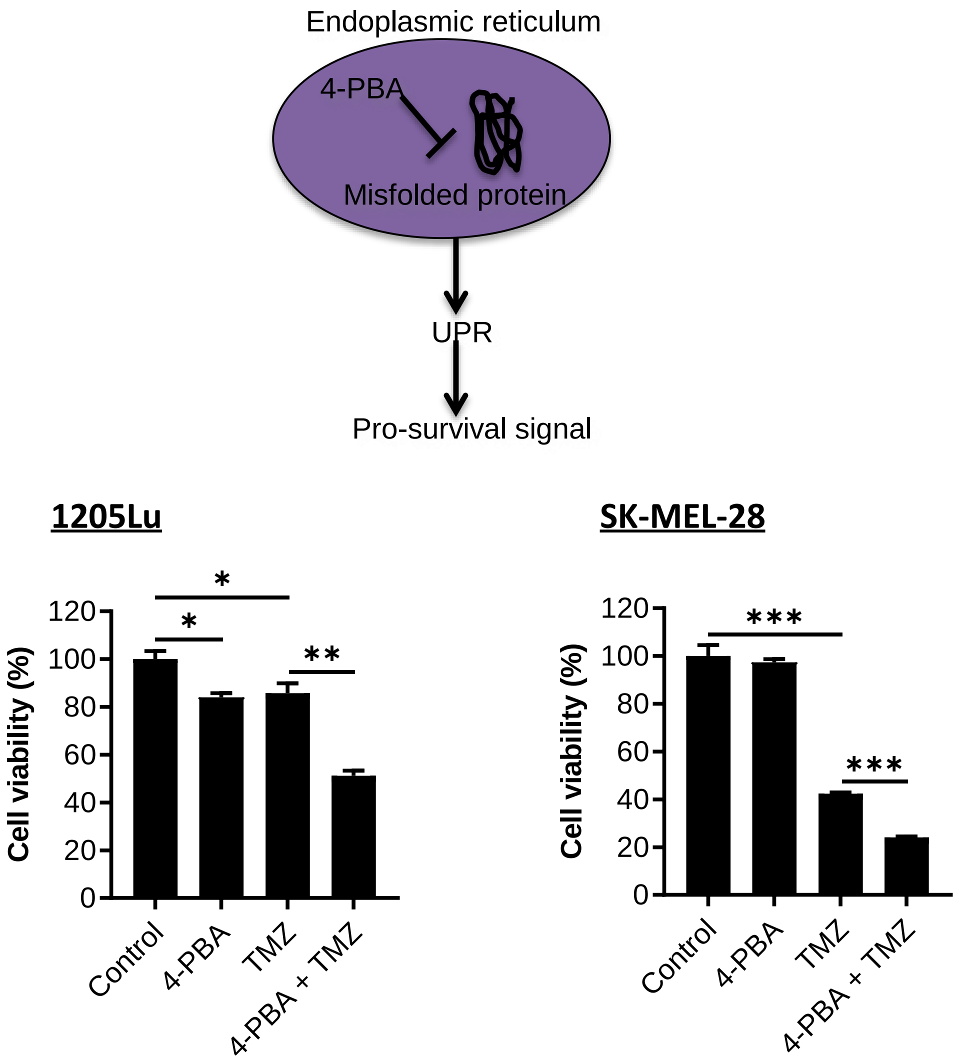

Figure S14. Pretreatment of 1205Lu and SK-MEL-28 cells with 4-phenylbutyric acid (PBA, 2 mM), an ER stress inhibitor, sensitizes cells to temozolomide (TMZ, 100 µM) and vemurafenib (VEM, 0.3 µM), respectively.

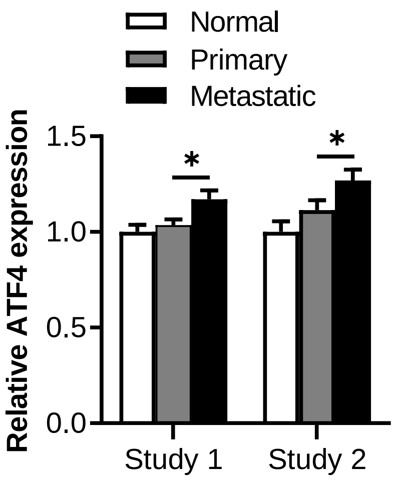

Figure S15. Metastatic melanoma tissues display higher *ATF4* mRNA level than primary melanoma by examining *ATF4* mRNA expression in human melanoma tissues using two publicly available microarray data sets.

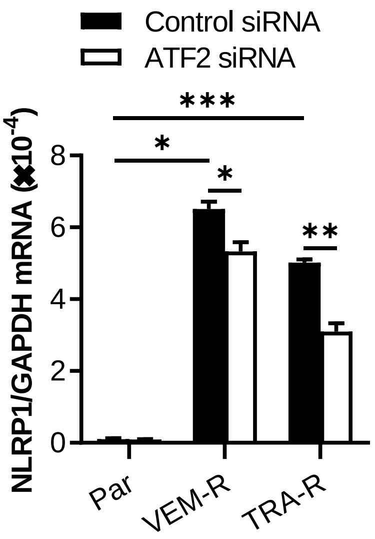

Figure S16. qRT-PCR analysis of *NLRP1* expression in parental and resistant SK-MEL-28 cells transfected with 50 nM control siRNA or ATF2 siRNA overnight. Mean ± SEM, n = 2-3. *p < 0.05, **p < 0.01, and ***p < 0.001.

Table S1. Primers used for qRT-PCR and ChIP.

Primers for qRT-PCR (SYBR green)

Gene	Gene ID	Forward	Reverse
IL1B	3553	CAGGCTGCTCTGGGATTCTC	GTCCTGGAAGGAGCACTTCAT
ATF4	468	ACCCAATTGGCCATCTCCCAGAA	ATCCACTTCACTGCCCAGCTCTAAAC
NLRP1	22861	GCTGGACCAGACAACTCTGA	GGTTTCCGTCTGCTGAAGAT
NLRP3	114548	GATCTTCGCTGCGATCAACAG	CGTGCATTATCTGAACCCCAC
MITF	4286	CAGGCATGAACACACATTCAC	TCCATCAAGCCCAAGATTTC
AXL	558	CAAGCACAGCCAGTCCACCA	GTTCAAACACTTCTCCATAACGGG
GAPDH	2597	CAGGGCTGCTTTTAACTCTGG	TGGGTGGAATCATATTGGAACA

Primers for ChIP (SYBR green)

Gene	Gene ID	Forward	Reverse
NLRP1	22861	TCTGCCAGTCCAAGTGAAACC	TCCTCTTTCGTGGAATCTGCAA
ATF3	467	TGAGTGAGACTGTGGCTGGGA	ATTGGTAACCTGGAGTTAAGCGGG
NLRP1*	22861	ACTCACTGCAACCTCCACCT	TGGTGAAACCCCGTCTCTAC

*2 kb upstream of the binding site
